# BAM 15 Exerts Molluscicidal Effects on *Pomacea canaliculata* Through the Induction of Oxidative Stress, Impaired Energy Metabolism, and Tissue Damage

**DOI:** 10.3390/molecules31020361

**Published:** 2026-01-20

**Authors:** Liping Wang, Haonan Yu, Guoli Qu, Jiankun Jin, Jie Wang, Yuntian Xing

**Affiliations:** 1 Wuxi School of Medicine, Jiangnan University, Wuxi 214122, China; 6242805011@stu.jiangnan.edu.cn; 2 National Health Commission Key Laboratory of Parasitic Disease Control and Prevention, Jiangsu Provincial Key Laboratory on Parasite and Vector Control Technology, Jiangsu Institute of Parasitic Diseases, Wuxi 214064, China; yuhaonan0403@163.com (H.Y.); quguoli@jipd.com (G.Q.); jk13032456395@163.com (J.J.); sunflower1230@163.com (J.W.)

**Keywords:** BAM 15, molluscicide, molluscicidal mechanism, *Pomacea canaliculata*, metabolomics, proteomics

## Abstract

Background: The golden apple snail (*Pomacea canaliculata*), an invasive species originating from South America, has inflicted considerable agricultural and ecological harm in non-native habitats. While the molluscicide niclosamide is currently effective against *P. canaliculata*, its prolonged use raises environmental concerns and the risk of resistance development. Results: BAM 15 possesses strong molluscicidal activity against *P. canaliculata*, with 72 h LC_50_ values of 0.4564 mg/L for adults (shell height: 20–25 mm), 0.3352 mg/L for subadults (10–15 mm), and 0.1142 mg/L for juveniles (2–3 mm). Metabolomic and proteomic profiling revealed that the altered metabolites and proteins both converged on energy metabolism and oxidative stress. Experimental validation revealed that BAM15 collapsed the mitochondrial membrane potential, drove MDA and H_2_O_2_ upward while depleting NADPH, boosted CAT, SOD and GPX activities, yet suppressed GR, and ultimately inflicted overt damage in the head-foot tissue of *P. canaliculata*. Conclusions: Our findings reveal that BAM 15 operates via a three-stage mechanism: (1) it disrupts membrane potential (ΔΨm) and impairs ATP production, severely disturbing energy metabolism; (2) energy deficits stimulate excessive electron transport chain activity, generating reactive oxygen species (ROS) and initiating oxidative stress; (3) persistent metabolic imbalance and oxidative damage culminate in extensive tissue injury. These results identify BAM 15 as a promising candidate for molluscicide development.

## 1. Introduction

The golden apple snail (*Pomacea canaliculata*), a freshwater snail species native to South America, is predominantly found in tropical and subtropical regions [1]. Introduced to East and Southeast Asia during the 1980s, this gastropod has since become a globally recognized invasive species due to its remarkable adaptability and absence of effective natural predators [2,3,4]. *P. canaliculata* not only inflicts severe damage on aquatic cash crops such as rice and lotus root but also preys on native aquatic fauna; consequently, its invasion leads to substantial ecological degradation across agricultural and aquatic ecosystems, including rice paddies, lakes, and wetlands [5,6].

In China, *P. canaliculata* is mainly concentrated in southern provinces including Zhejiang, Shanghai, and Fujian. However, rising global temperatures have facilitated its rapid population growth and contributed to the northward expansion of its geographic range [7,8]. Consequently, there is an increasingly urgent need for strategies that can enable the more effective management of this invasive species. Current control measures include biological, physical, and chemical approaches. Biological methods have had limited success to date in this context. For example, although ducks are known predators, they rarely consume *P. canaliculata* eggs owing to digestive limitations [9]. Physical strategies, such as manual collection, are labor-intensive and inefficient. As a result, chemical control through the use of molluscicides has become the dominant approach to controlling the spread of this snail species. Niclosamide (NIC) remains the only molluscicide endorsed by the World Health Organization (WHO) in this context owing to its high efficacy against snails and low toxicity to mammals [10]. However, the exclusive and prolonged application of NIC raises concerns over resistance development in *P. canaliculata* populations [11]. This highlights the urgent need for novel molluscicidal agents with distinct structures and mechanisms of action.

BAM 15 is a compound that has been extensively studied for its broad biological effects, including anti-obesity [12], glucose regulation [13], anti-inflammatory activity [14], and ability to enhance mitochondrial redox capacity [15]. Both in vitro and in vivo studies have demonstrated its low mammalian toxicity and favorable safety profile [16,17]. Despite this, the molluscicidal potential of BAM 15 has not been previously evaluated. In this study, we investigated the molluscicidal activity of BAM 15 against *P. canaliculata*, assessed its efficacy across developmental stages, and explored its underlying mechanism using integrated metabolomic and proteomic analyses, supported by biochemical validation. Our findings offer a valuable addition to current molluscicidal strategies and provide a novel structural scaffold as an option for the development of snail control agents.

## 2. Results

### 2.1. Molluscicidal Activity

BAM 15 demonstrated robust molluscicidal activity against *P. canaliculata*, LC_50_ values for juvenile snails (2–3 mm shell height) were 0.1336, 0.1142, and 0.1142 mg/L at 24, 48, and 72 h, respectively. For subadults (10–15 mm), LC_50_ values were 0.5860, 0.3352, and 0.3352 mg/L over the same time intervals. Adult snails (20–25 mm) exhibited LC_50_ values of 0.7365 mg/L at 24 h, decreasing to 0.4625 mg/L at 48 h and 0.4564 mg/L at 72 h (Table 1).

### 2.2. Metabolomic Analyses

**Figure 1:**
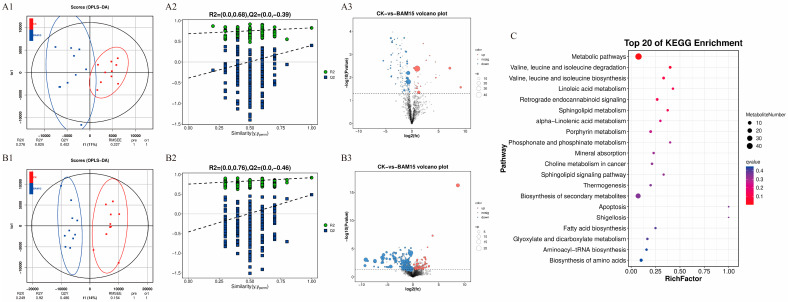
Identification, cluster analysis, and pathway enrichment of differentially expressed metabolites in *P. canaliculata* soft tissues. Note: (**A1**): OPLS-DA score plot in NEG mode; (**A2**): OPLS-DA permutation test plot in NEG mode; (**A3**): Volcano plot in NEG mode; (**B1**): OPLS-DA score plot in POS mode; (**B2**): OPLS-DA permutation test plot in POS mode; (**B3**): Volcano plot in POS mode; (**C**): KEGG enrichment pathway map.

A total of 282 differentially abundant metabolites (DAMs) were detected in BAM 15-treated snails, with 72 showing upregulation and 210 displaying downregulation relative to controls (Figure 1(A3,B3)). Kyoto Encyclopedia of Genes and Genomes (KEGG) enrichment analysis revealed that these metabolites were implicated in metabolic pathways; valine, leucine, and isoleucine degradation; and valine, leucine, and isoleucine biosynthesis (Figure 1C). The OPLS-DA score plots showed distinct clustering between treatment and control groups, indicating significant metabolic divergence (Figure 1(A1,B1)). Permutation testing confirmed the validity of the model, with Q^2^ intercepts below zero (Figure 1 (A2,B2)).

Changes in the levels of some metabolites revealed metabolic disturbances in *P. canaliculata*, including elevated levels of ADP and hydroxyurea, as well as reduced levels of trilaurin, glycerol, glycine, L-leucine, tartaric acid, and glycolate (Table 2).

### 2.3. Proteomic Analyses

**Figure 2:**
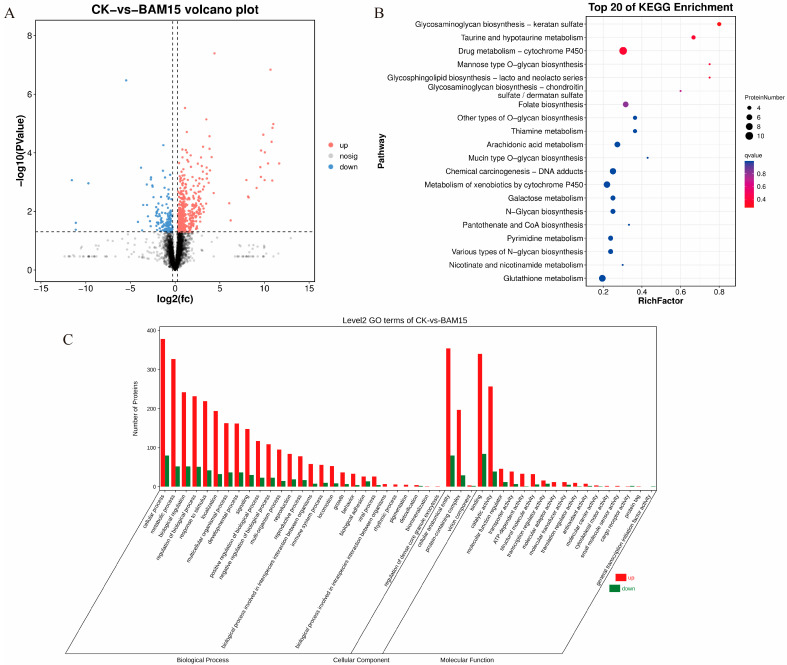
Identification and enrichment analysis of differentially expressed proteins (DEPs) in *P. canaliculata* soft tissues. Note: (**A**): Volcano plot of differential proteins; (**B**): GO enrichment plot; (**C**): KEGG enrichment plot.

Proteomic analysis identified 617 significantly differentially expressed proteins (DEPs) in the BAM 15-treated group compared to controls, comprising 474 upregulated and 143 downregulated proteins (Figure 2A). Gene Ontology (GO) enrichment analysis revealed significant involvement in 27 biological process terms (e.g., metabolic processes, biological regulation), 3 cellular component terms (e.g., anatomical structures and protein-containing complexes), and 17 molecular function terms (e.g., catalytic activity and binding) (Figure 2B). KEGG pathway analysis showed enrichment in glycosaminoglycan biosynthesis–keratan sulfate, taurine and hypotaurine metabolism, and cytochrome P450-mediated drug metabolism pathways (Figure 2C).

We focused on a subset of DEPs whose alterations exhibited strong concordance with metabolomic predictions, including adenylate kinase (AK), aldo-keto reductase family 1 member B (AKR1B), carbonyl reductase 1 (CBR1), flavin-containing monooxygenase (FMO), glutathione S-transferase (GST), monoamine oxidase (MAO), core 1 β1,3-galactosyltransferase 1 (CGNT1), and polypeptide *N*-acetylgalactosaminyltransferase (GALNT). The expression levels of these proteins were all significantly up-regulated (Table 3).

### 2.4. Mitochondrial Membrane Potential

**Figure 3:**
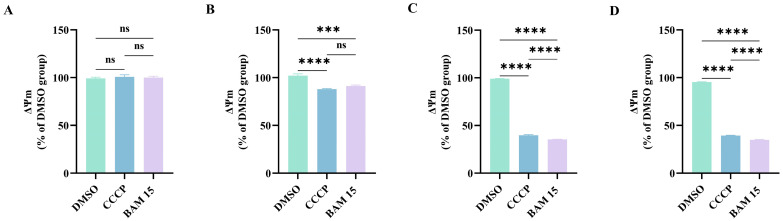
Effects of BAM 15 on mitochondrial membrane potential (ΔΨm) in *P. canaliculata*. Note: (**A**): ΔΨm prior to drug treatment; (**B**): ΔΨm immediately after drug addition; (**C**): ΔΨm at 15 min post-drug treatment; (**D**): ΔΨm at 30 min post-drug treatment. Data are shown as mean ± SD, with *n* = 3 biological replicates. ns: not significant, *** *p* < 0.001, **** *p* < 0.0001.

Exposure to BAM 15, along with the known mitochondrial uncoupler carbonyl cyanide m-chlorophenyl hydrazone (CCCP, positive control), led to a rapid and significant decline in ΔΨm compared to the DMSO control (*p* < 0.0001). This decline intensified over 15 and 30 min intervals, with BAM 15 demonstrating a more pronounced and sustained effect than CCCP by the 30 min mark (*p* < 0.0001) (Figure 3).

### 2.5. Effects of BAM 15 on Oxidative Stress Biomarkers in P. canaliculata

**Figure 4:**
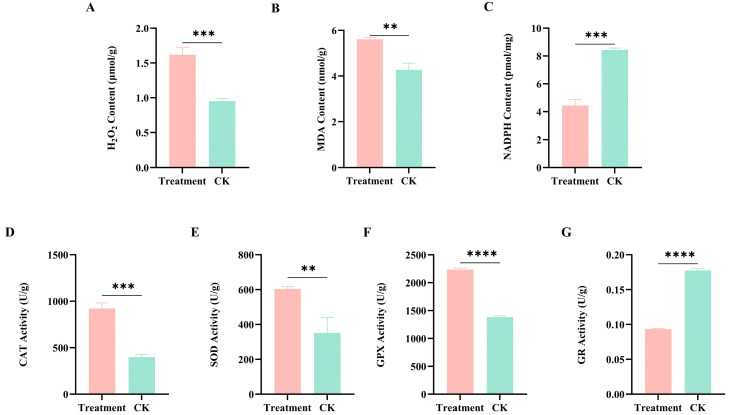
Effects of BAM 15 on oxidative stress-related biochemical parameters in *P. canaliculata*. Note: (**A**): H_2_O_2_ content; (**B**): MDA content; (**C**): NADPH content; (**D**): CAT activity; (**E**): SOD activity; (**F**): GPX activity; (**G**): GR activity. Data are shown as mean ± SD, with *n* = 3 biological replicates. ** *p* < 0.01, *** *p* < 0.001, **** *p* < 0.0001.

After 24 h of exposure to BAM 15, MDA and H_2_O_2_ levels in *P. canaliculata* tissues were significantly increased, while NADPH was markedly reduced. In response, CAT, SOD, and GPX antioxidant enzyme activity levels were significantly elevated. Conversely, GR activity was significantly suppressed (Figure 4).

### 2.6. Histopathological Changes

**Figure 5:**
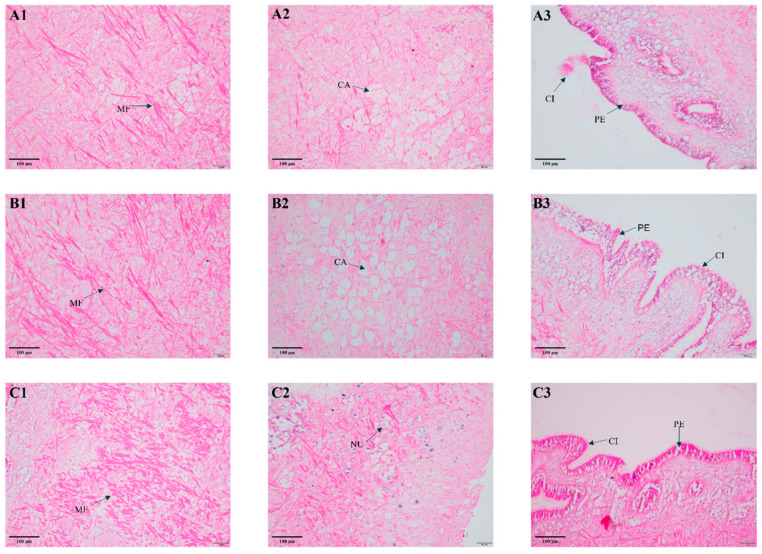
Comparison of soft tissues morphology following treatment with BAM 15. Note: (**A1**–**A3**): Treatment with 1/2 LC_50_ (24 h) of BAM 15; (**B1**–**B3**): Treatment with LC_50_ (24 h) of BAM 15; (**C1**–**C3**): Treatment with dechlorinated water supplemented with 0.1% (*v*/*v*) DMSO (control). MF: Muscle fibers; CA: Cavities; CI: Cilia; PE: Pedal epithelium; NU: Nuclei.

The soft tissue (foot) of *P. canaliculata* is composed of three structural layers: an outer pseudostratified ciliated columnar epithelium, underlying connective tissue, and an inner muscular layer. In untreated specimens, muscle fibers appeared dense and regularly organized (Figure 5(C1)), with clearly identifiable nuclei (Figure 5(C2)) and intact mucosal architecture, including a smooth, ciliated epithelial surface (Figure 5(C3)). In contrast, BAM 15 treatment caused pronounced morphological damage. Muscle fibers became markedly thinner (Figure 5(A1,B1)), with widespread cellular necrosis and nuclear disappearance (Figure 5(A2,B2)). The mucosal cilia showed aggregation and detachment, and at the higher concentration (LC_50_ of 24 h), the mucosal epithelium displayed significant fissuring and sloughing (Figure 5(A3,B3)).

## 3. Discussion

The impact of *P. canaliculata* on the native ecosystem is multifaceted and profound [18]. NIC remains the only molluscicide formally endorsed by the WHO, yet continued reliance on this single chemotype inevitably drives resistance in *P. canaliculata.* Consequently, the discovery and development of novel, mechanistically distinct molluscicidal agents are now a high-priority imperative for sustainable snail management. In present study, we found that BAM 15 has robust molluscicidal activity against *P. canaliculata*, exhibiting a clear dose- and time-dependent relationship across all developmental stages (Table 1). Comparative analysis of the 72 h LC_50_ values of BAM 15 across different size classes of snails revealed that juvenile individuals exhibited significantly higher sensitivity to the compound, with susceptibility approximately 2.94- and 4.00-fold greater than that of subadult and adult snails, respectively. This would be beneficial in field application. After dissipation for several days, while the residual concentration of BAM 15 may be insufficient to kill adults, it would probably be sufficient to kill juveniles and would thus control population growth [19].

To clarify the molluscicidal mechanism of action exhibited by BAM 15, we employed a comprehensive omics strategy to investigate molecular alterations in adult *P. canaliculata* following exposure to sublethal concentration (1/2 LC_50_ of 24 h) of BAM 15. In the metabolomic analysis, we surmised that changes in the levels of certain metabolites might be related to metabolic disorder in *P. canaliculata* (Table 2). These DAMs were primarily enriched in fundamental metabolic networks, including the degradation and biosynthesis pathways of branched-chain amino acids (valine, leucine, and isoleucine). These pathways are integral to energy production and amino acid homeostasis: the catabolic of valine/leucine/isoleucine generates energy via intermediates such as acetyl-CoA, while their biosynthetic pathways maintain protein synthesis. BAM 15 exposure therefore appears to disrupt both energy-generating and anabolic processes, impairing metabolic function and physiological stability in *P. canaliculata*. Mechanistically, the observed increase in ADP levels suggests energy synthesis impairment, likely triggering compensatory shifts to alternative energy sources. These include (i) L-leucine entry into the TCA cycle through branched-chain amino acid degradation to produce acetyl-CoA [20]; (ii) β-oxidation of free fatty acids derived from trilaurin [21]; and (iii) engagement of glycerol and glycine in gluconeogenic pathways [22,23,24]. Simultaneously, hydroxyurea accumulation may intensify oxidative stress by promoting reactive oxygen species (ROS) generation [25], while the depletion of endogenous antioxidants including L-leucine [26,27], glycine [28], glycolate [29], and tartaric acid [30,31,32] possibly due to its involvement in the organism’s antioxidant processes. Reduced L-leucine levels may also impair protein synthesis [33] and the repair and growth of muscle tissue [34], exacerbating tissue damage.

Proteomic profiling showed enrichment in glycosaminoglycan biosynthesis–keratan sulfate, taurine and hypotaurine metabolism, and cytochrome P450-mediated drug metabolism pathways (Figure 2C). Keratan sulfate, a complex mucin-associated glycosaminoglycan, contributes to epithelial protection, while taurine and hypotaurine metabolism are pivotal for lipid regulation and oxidative stress modulation. The cytochrome P450 pathway, crucial for xenobiotic and endogenous compound metabolism, reflects heightened detoxification activity. Specifically, we focused on a subset of DEPs potentially linked to the mechanism of action of BAM 15 (Table 3). AK catalyzes the reversible conversion of 2ADP ↔ ATP + AMP [35]; given the elevated ADP pool observed, we infer that *P. canaliculata* intensifies ATP salvage pathways to sustain cellular energy homeostasis under stress. In oxidative defense, AKR1B detoxifies reactive carbonyl species and mitigates lipid peroxidation [36], while CBR1 counters ROS-induced cellular damage [37]. These enzymes represent key antioxidant defenses mobilized under BAM 15-induced stress. Together with the remodeling of antioxidant metabolites, we conclude that BAM 15 elicited oxidative stress in *P. canaliculata*, prompting transcriptional up-regulation of antioxidant enzymes while concomitantly consuming antioxidant metabolites to preserve cellular redox homeostasis. Additionally, FMO, GST, and MAO collectively contribute to chemical detoxification through oxidative metabolism [38], glutathione conjugation [39,40], and amine clearance [41]. Moreover, increased expression of CGNT1 and GALNT suggests enhanced mucin biosynthesis, potentially fortifying the physical barrier against environmental stressors [42,43]. This multifaceted molecular response demonstrates the adaptive resilience of *P. canaliculata* in response to BAM 15 exposure and may partially explain the species’ high environmental adaptability.

Integrated omics analyses have essentially established that BAM 15 exposure evokes systemic physiological disruption in *P. canaliculata*, encompassing impaired energy metabolism, redox imbalance, and perturbed muscle-protein homeostasis. Nevertheless, dedicated experimental validation is still required. Firstly, we assessed ΔΨm—a key indicator of ATP synthesis capacity—to validate that BAM 15 compromises energy metabolism in *P. canaliculata* (Figure 3). In mammalian systems, BAM 15 has been shown to disrupt the proton gradient across the inner mitochondrial membrane, leading to ΔΨm dissipation and impaired ATP production [44]. Our investigation confirmed that similar uncoupling effects occur in mollusks.

Under conditions of cellular energy deprivation, compensatory hyperactivation of the mitochondrial electron transport chain occurs in an attempt to sustain ATP synthesis [45]. As the electron transport chain is a primary intracellular source of ROS, such upregulation inevitably increases ROS production, ultimately leading to widespread oxidative stress [46,47]. However, the specific role of BAM 15 in modulating ROS remains uncertain, with various suggestions that it promotes ROS biogenesis or impairs scavenging activity [48,49,50]. To clarify this mechanistic ambiguity, we measured key biochemical markers of oxidative stress, including levels of H_2_O_2_, MDA, and NADPH, as well as the activity of the key antioxidant enzymes SOD, CAT, GR, and GPX (Figure 4). This rise in the content of H_2_O_2_, as a central ROS molecule, provides direct confirmation of the overall enhancement of ROS accumulation. Higher levels of MDA, which is a well-established end-product of lipid peroxidation and a sensitive indicator of oxidative membrane damage, further indicate the occurrence of oxidative injury in lipid-rich tissues [51]. Under normal physiological conditions, NADPH is primarily synthesized via the pentose phosphate pathway (PPP) and functions as a key reducing agent that fuels antioxidant defense systems [52]. However, under BAM 15-induced stress, ATP shortage caused by uncoupling presumably redirected glucose flux from the pentose-phosphate pathway to glycolysis, prioritizing ATP generation over redox balance. This metabolic reprogramming, together with rising ROS levels, likely accounts for the observed decline in NADPH content. Interestingly, BAM 15 induced differential regulation of antioxidant enzymes. Activities of CAT, SOD, and GPX were markedly upregulated, suggesting a compensatory response aimed at counteracting oxidative imbalance and preserving cellular redox equilibrium [53]. In contrast, GR, an NADPH-dependent enzyme, showed significantly reduced activity, likely attributable to altered NADPH availability. Although GR and GPX typically exhibit coordinated activity to maintain intracellular glutathione homeostasis, divergent responses can emerge under specific conditions. For instance, Hao et al. have demonstrated that GR suppression triggers compensatory upregulation of GPX activity to mitigate excess ROS, which is consistent with our results [54].

**Figure 6:**
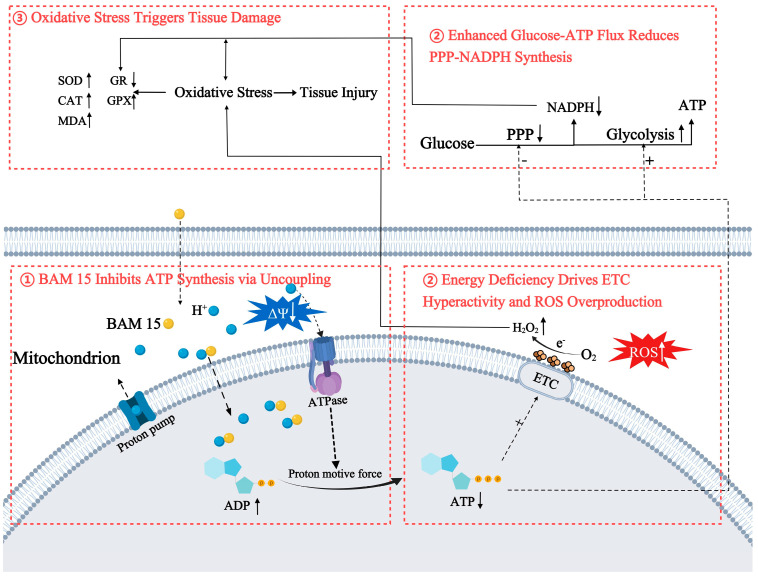
The molluscicidal mechanism of BAM 15.

Given the established link between oxidative stress and tissue injury [55,56], we investigated the structural integrity of soft tissues in *P. canaliculata* following BAM 15 exposure using histological sectioning and microscopic examination. Our analysis revealed substantial morphological abnormalities in BAM 15-treated individuals (Figure 5). The observed thinning of muscle fibers may reflect disrupted protein synthesis and impaired muscle maintenance, aligning with our metabolomics-based analysis. These histopathological signs provide an evidence that BAM 15 elicits tissue-level toxicity, likely mediated through oxidative stress. Collectively, our data delineate the integrated mechanism of action of the compound (Figure 6).

It is important to note that while this study establishes the potent molluscicidal activity and mechanistic basis of BAM 15 against *P. canaliculata*, its potential environmental and agricultural safety profile remains to be fully evaluated. Given that mitochondrial uncoupling is a fundamental biological process conserved across eukaryotes, a comprehensive assessment of BAM 15’s ecotoxicological profile is an essential prerequisite for its consideration as an agrochemical. Future research must prioritize rigorous toxicity testing on a range of representative non-target species to determine its selectivity and environmental safety before field application.

## 4. Materials and Methods

### 4.1. Snails and BAM 15 Preparation

A laboratory-maintained colony of *P. canaliculata* was established in 2021, reared in plastic containers (dimensions: 40 cm × 45 cm × 40 cm) containing dechlorinated water (pH 7.2 ± 0.2, dissolved oxygen 6.0 ± 0.4 mg L^−1^, hardness 68–78 mg L^−1^ as CaCO_3_) under a 12 h light:12 h dark cycle at 25 ± 2 °C [57]. Approximately 50 snails were stocked per container, maintained on a daily ration of fresh lettuce leaves with twice-weekly water replacement prior to testing. Individuals used in the bioassay were randomly selected and food was withheld for the entire exposure period [58]. The test compound, BAM 15, was obtained from MedChemExpress (Monmouth Junction, NJ, USA) and dissolved in dimethyl sulfoxide (DMSO) to prepare a 5 mg/mL stock solution.

### 4.2. Evaluation of Molluscicidal Efficacy

Serial dilutions of BAM 15 were prepared in dechlorinated water to yield concentrations of 1.0, 0.5, 0.25, 0.125, and 0.0625 mg/L. Snails were sorted into three groups based on shell height: juveniles (2–3 mm), subadults (10–15 mm), and adults (20–25 mm). For each replicate, 10 snails were transferred to a 500 mL glass beaker containing either 500 mL of the test solution or 500 mL of dechlorinated water supplemented with 0.1% (*v*/*v*) DMSO (control). Snails were fully submerged beneath a stainless-steel mesh screen that maintained uniform contact with the medium while preventing escape. Mortality was monitored continuously and recorded at 24, 48 and 72 h under a 12 h light: 12 h dark photoperiod at 25 ± 2 °C, with death confirmed by the absence of a reflex response to mechanical probing with a stainless steel needle, followed by the removal of deceased individuals. Each treatment was replicated three times.

### 4.3. Omics Analyses

Twenty adult snails were randomly assigned to two groups (*n* = 10/group). The treatment group was exposed to 1/2 50% lethal concentration (LC_50_, 0.7365 mg/L) of BAM 15 (24 h) [59], while the control group received dechlorinated water supplemented with 0.1% (*v*/*v*) DMSO. After 24 h of exposure, soft tissues were collected, immediately snap-frozen in liquid nitrogen, and stored at −80 °C until further processing for metabolomic and proteomic analyses.

### 4.4. Metabolomic Analysis

For metabolomics, tissues (250 mg per snail, *n* = 10/group) were quenched in liquid nitrogen, pulverised, and extracted with 1 mL ice-cold methanol:water (4:1, *v*/*v*) containing 10 µg/mL 2-chloro-L-phenylalanine as internal standard. After vortexing and centrifugation (12,000× *g,* 4 °C, 15 min), 100 µL aliquots of each supernatant were pooled to generate a quality control (QC) sample; blanks consisted of extraction solvent only. Samples were randomised prior to injection and analysed on a Waters ACQUITY UPLC I-Class (Milford, MA, USA) coupled to a SYNAPT G2-Si quadrupole time-of-flight operated in positive/negative (POS/NEG) electrospray ionisation mode (capillary 2.5 kV, source 120 °C, desolvation 350 °C, cone 30 V). Data were acquired (*m*/*z* 50–1000, 0.2 s/scan) and processed with Progenesis QI (v.2.3) for peak picking (gradient 5–95% B over 12 min, alignment tolerance 0.5 min), deconvolution, and normalisation to total ion intensity. Missing values (<20% across samples) were imputed by k-nearest-neighbour (k-NN, k = 5); features with coefficient of variation >30% in QCs or present in <50% of either group were excluded prior to multivariate statistics.

### 4.5. Roteomic Analysis

For proteomics, mitochondria-enriched fractions were isolated from pooled soft tissues (100 mg per snail, *n* = 5/group) using a commercial kit, lysed in 1% sodium deoxycholate/8 M urea with protease inhibitors, and quantified (via bicinchoninic acid protein assay). After iST kit-based reduction, alkylation and trypsin digestion, peptides were fractionated by high-pH reversed-phase chromatography (Waters nanoACQUITY, 2 µL/min, 4–68% B in 70 min) and analysed by data-independent acquisition (DIA) nanoLC-tandem mass spectrometry (MS/MS) (UltiMate 3000, Thermo Fisher Scientific, Waltham, MA, USA, coupled to a timsTOF Pro^2^, Bruker Daltonics, Billerica, MA, USA. 400 nL/min, 60 min gradient, 22 × 40 Th windows, *m*/*z* 349–1229). A hybrid data-dependent acquisition (DDA) library was generated in Spectronaut 18 (1% false discovery rate, FDR; carbamidomethyl-C fixed, methionine oxidation and N-acetyl variable) and used for DIA quantification (MaxLFQ, 1% precursor & protein FDR, local normalisation). Peptides with Q-value < 1% were retained; proteins detected in <2 replicates per group were filtered out before statistical testing.

### 4.6. Mitochondrial Membrane Potential Analyses

Mitochondria were isolated from five adult snails using a mitochondrial isolation kit, and the resulting mitochondrial suspensions were combined for downstream analyses. Following determination of protein concentration, mitochondrial membrane potential (ΔΨm) was assessed before and after 1/2 LC_50_ (24 h) of BAM 15 treatment using JC-1 dye, with fluorescence measured using a microplate fluorometer. Kits for mitochondrial isolation, protein quantification, and ΔΨm detection were procured from Shanghai Biyuntian Biological Co., Ltd. (Shanghai, China).

### 4.7. Evaluation of Oxidative Stress-Associated Biomarkers

After 24 h of exposure to 1/2 LC_50_ (24 h) of BAM 15, soft tissues of three adult snails were carefully separated on ice. A precise 0.1 g of tissue was homogenized in 1 mL of the corresponding extraction buffer, and the supernatants were used for biochemical assays. Markers including malondialdehyde (MDA), hydrogen peroxide (H_2_O_2_), and nicotinamide adenine dinucleotide phosphate (NADPH), as well as the enzymatic activities of catalase (CAT), glutathione reductase (GR), superoxide dismutase (SOD), and glutathione peroxidase (GPX), were measured using assay kits provided by Beijing Solarbio Science & Technology Co., Ltd. (Beijing, China).

### 4.8. Histopathology

Histological examinations of adult snails were performed following exposure to 1/2 LC_50_ (24 h) and LC_50_ (24 h) of BAM 15 for 24 h (*n* = 5/group). Post-treatment, snail soft tissues were fixed in 4% paraformaldehyde and subjected to a graded ethanol dehydration series. Samples were then cleared in xylene and embedded in paraffin wax. Using a rotary microtome, sections were cut at a thickness of 5 µm, with three replicate slices prepared for each tissue sample. Subsequently, the sections were stained with hematoxylin and eosin (H&E) to facilitate microscopic observation. Histological features were evaluated under a light microscope according to established protocols [60].

### 4.9. Data Analysis

Differential metabolites (variable importance in projection, VIP > 1; *p* < 0.05 from two-tailed Student’s *t*-test) were retained only after Benjamini–Hochberg false-discovery rate (FDR) < 0.05. Orthogonal partial least squares discriminant analysis (OPLS-DA) metrics: R^2^X, R^2^Y, Q^2^ ≥ 0.4; cross-validated analysis of variance (CV-ANOVA) and 200-iteration permutation *p* < 0.05. Differentially expressed proteins (fold-change > 1.2, *p* < 0.05 from two-tailed Student’s *t*-test) were submitted to Benjamini–Hochberg FDR < 0.05. Peptide and protein identifications were filtered at 1% FDR in Spectronaut. Principal component analysis (PCA) of samples showed replicate correlation R > 0.92.

For molluscicidal activity and biochemical assay data, analyses were performed using GraphPad Prism v9.5. Molluscicidal potency was expressed as LC_50_ with 95% confidence intervals (CI) determined by non-linear regression (log(inhibitor)vs. normalized response—Variable slope). ΔΨm and oxidative-stress biomarkers are reported as mean ± standard deviation (SD). ΔΨm data were analysed by One-way ANOVA followed by Tukey’s multiple comparisons test. Oxidative-stress biomarkers were compared between two groups with the unpaired two-tailed Student’s *t*-test after confirming normal distribution by Shapiro–Wilk test. Differences were considered statistically significant at *p* < 0.05.

## 5. Conclusions

In summary, the present results reveal that BAM 15 exerts strong molluscicidal effects against *P. canaliculata* across various developmental stages. Mechanistically, its action can be attributed to three central interrelated effects: (1) BAM 15 disrupts ΔΨm, leading to impaired ATP synthesis; (2) the resulting energy deficit intensifies electron transport chain activity, amplifying ROS production and initiating oxidative stress; and (3) persistent redox imbalance, coupled with metabolic dysregulation, culminates in structural damage to muscle tissue. These findings not only offer new insight into the processes by which BAM 15 impairs physiological homeostasis in *P. canaliculata* but also highlight its potential utility as a novel molluscicidal agent. Future studies will focus on assessing the environmental safety and ecological impacts of BAM 15.

## Figures and Tables

**Table 1:** Molluscicidal efficacy of BAM 15 against *P. canaliculata*.

Treatment Time (h)	Diameter of Snails (mm)	LC_50_ (mg/L)	Toxicity Equation	R Squared	LC_50_ (mg/L, 95%CI)
24	2–3	0.1336	Y = 100/(1 + 10^(−0.8743 − x) × 5.779^)	0.9952	0.1230–0.1479
	10–15	0.5860	Y = 100/(1 + 10^(−0.2321 − x) × 2.321^)	0.9797	0.4636–0.7629
	20–25	0.7365	Y = 100/(1 + 10^(−0.1329 − x) × 1.842^)	0.9637	0.5438–1.147
48	2–3	0.1142	Y = 100/(1 + 10^(−0.9424 − x) × 4.703^)	0.9989	0.1086–0.1195
	10–15	0.3352	Y = 100/(1 + 10^(−0.4746 − x) × 3.617^)	0.9985	0.3114–0.3612
	20–25	0.4625	Y = 100/(1 + 10^(−0.3349 − x) × 3.441^)	0.9884	0.3893–0.5572
72	2–3	0.1142	Y = 100/(1 + 10^(−0.9424 − x) × 4.703^)	0.9989	0.1086–0.1195
	10–15	0.3352	Y = 100/(1 + 10^(−0.4746 − x) × 3.617^)	0.9985	0.3114–0.3612
	20–25	0.4564	Y = 100/(1 + 10^(−0.3407 − x) × 4.060^)	0.9986	0.4306–0.4824

**Table 2:** Several differentially abundant metabolites (DAMs) associated with BAM 15 exposure.

Compound ID	Formula	Name	CAS Number	Type
M428T489_POS	C_10_H_15_N_5_O_10_P_2_	ADP	58-64-0	up
M662T173_POS	C_39_H_74_O_6_	Trilaurin	538-24-9	down
M91T35_NEG	C_3_H_8_O_3_	Glycerol	56-81-5	down
M75T42_NEG	C_2_H_4_O_3_	Glycolate	79-14-1	down
M76T327_POS	C_2_H_5_NO_2_	Glycine	56-40-6	down
M130T184_NEG	C_6_H_13_NO_2_	L-Leucine	61-90-5	down
M77T272_POS	CH_4_N_2_O_2_	Hydroxyurea	127-07-1	up
M149T37_NEG	C_4_H_6_O_6_	Tartaric acid	87-69-4	down

**Table 3:** Several differentially expressed proteins (DEPs) associated with BAM 15 exposure.

ID	Name	Symbol	Type
K00939	Adenylate kinase	AK	Up
K00011	Aldose reductase	AKR1B	Up
K00079	Carbonyl reductase 1	CBR1	Up
K00485	Hypotaurine monooxygenase	FMO	Up
K00274	Monoamine oxidase	MAO	Up
K00799	Glutathione S-transferase	GST	Up
K00710	Polypeptide *N*-acetylgalactosaminyltransferase	GALNT	Up
K00727	β-1,3-galactosyl-*O*-glycosyl-glycoprotein β-1,6-*N*-acetylglucosaminyltransferase	GCNT1	Up

## Data Availability

The original contributions presented in this study are included in the article. Further inquiries can be directed to the corresponding author.
